# Progress in Psychiatry

**Published:** 1903-02-28

**Authors:** 


### Progress in Psychiatry.

(*Concluded from page* 340.)

**Heredity of Mental Disease : Special Problems.**—The question as to the greater potency of one or other parent in transmitting a tendency to insanity is one on which opinions differ. Esquirol was of opinion that an insane mother was more potent as a transmitter of the taint than an insane father, a view confirmed by Baillarger [8] and others, and generally assumed to be correct. The cases studied by Turner [9] agree with the above in showing the greater potency of maternal transmission. Farquharson, however, finds the maternal and paternal factors practically equal, the proportions being as 8·2 (maternal) to 8·1 (paternal), and this practical equality is also borne out by Dr. Wiglesworth's figures which are 9·11 (maternal) and 8·88 (paternal). The conclusion is therefore reached that the female sex, as such, has little if any greater power of transmitting insanity than the male sex. The relative potency of either parent in transmitting the taint of insanity is governed by natural laws of which we are still profoundly ignorant, and which stand in urgent need of elucidation. Another point of great interest is whether the insanity of the parent bears any relation to the sex of the child—that is to say, whether either parent has a greater tendency to transmit to the children of his or her own sex. The statistics collected by Baillarger, Thurnam,[10] Brigham,[11] Grainger Stewart, and Farquharson, point to the view that the father's insanity was somewhat more liable to be transmitted to the sons, whilst the mother had a greater tendency to transmit the disease to the daughters. Turner, however, found that whichever parent was insane, the daughters were more often affected than the sons, and that this was more frequently the case when the mother was insane. Dr. Wiglesworth's own statistics harmonised with those of Turner. Thus from a total of 306 cases of insanity in which the paternal influence was paramount, 130 of the offspring were males and 176 females. Again from a total of 314 instances in which the maternal influence was paramount, 124 were males and 190 females. While there is thus some doubt as to whether the father's insanity tends to pass with greater frequency to the male than the female offspring, there is a unanimous consensus of statistics and of opinion (Baillarger, Thurnam, Brigham, Grainger Stewart, Farquharson, Turner, Wiglesworth) as to the tendency of the mother to transmit with greater frequency to the female than to the male offspring. Turner has suggested that the relative influence of either parent upon the offspring of the corresponding sex could be best ascertained by eliminating from inquiry all cases of insanity commencing in adult life, and confining the inquiry to congenitals and adolescents only, so as to eliminate as far as practicable all extraneous causes of the disease. Proceeding in this way he found that more imbecile sons than daughters were born to insane fathers, and more imbecile daughters than sons to insane mothers. The number of cases of congenital idiocy and imbecility studied by Dr. Wiglesworth include 68, and of these 35 were males and 33 females. While the proportion of hereditary taint was 37·14 per cent. for the male cases it was 51·51 per cent. for the female cases. The number of cases of epileptic insanity (not including epileptic idiocy and epileptic imbecility) studied was 120, and of these 77 were males and 43 females. The male epileptics showed 19·48 as having a hereditary origin while the female epileptics gave a proportion of 53·48 per cent. Part of this great disparity may be due, says Dr. Wiglesworth, to accidental causes, and it may be that if larger numbers are taken the real disparity would be found not so great. Still, the figures tend to show that epilepsy in the male is far more of an acquired affection than it is in the female. The number of cases of general paralysis observed was 433, of which 363 were males and 70 females, and out of these 82 (60 males and 22 females) showed a definite family history of insanity, which gives a percentage on the total number of 18·93. Farquharson's statistics give a percentage of 18·6, based on a study of 231 cases. Combining these figures we have a grand total of 664 cases of general paralysis, of which 18·8 per cent. gave a definite family history of insanity. On dividing the cases into sexes, however, it is found that female cases of general paralysis reveal a history of insanity in the parentage nearly twice as often proportionately as male cases, the figures being as 31·4 to 16·5. The unequal incidence of heredity in the two sexes points to general paralysis being much more of an acquired affection in the male than in the female. Other forms of insanity are, by reason of faulty present-day classification, not correctly capable of statistical treatment so as to give trustworthy results. The study of idiocy and of imbecility (both non-epileptic and epileptic forms), of epileptic insanity, and of general paralysis, all of which are clearly recognisable types of insanity, show that female patients reveal the taint of morbid ancestry almost twice as often as male patients. [Dr. Wiglesworth cites interesting considerations regarding the hereditary sequelæ of alcoholism and of syphilis which will be referred to later.] There always remains, however a fair minority of insane patients in asylums as regards whom an antecedent family taint of insanity cannot be traced. Moreover, cases are from time to time met with where a strong direct taint of insanity exists in a family, and yet some at least of the offspring escape being tainted and grow up mentally sound. Finally we occasionally meet with cases of insanity which apparently arise *de novo* where, the family records being good, we should not expect to meet with cases of this affection, and *per contra* even when both parents are affected with insanity a small proportion of the offspring escape the disease. "Doubtless in many of these cases," adds Dr. Wiglesworth, "the variations in the germ plasm which have culminated in the insanity of the parents have been of recent development, and hence . . . would be less likely to be handed down in all their fulness than if they had gathered strength by transmission through a long line of ancestors." We have an illustration in such cases of that "regression towards the mean standard" of the race which Galton[12] has called attention to, and which he has worked out in its application to the stature of the population. "The law of regression tells heavily against the full hereditary transmission of any gift." And this law applies equally to the handing on of good as of bad qualities, of sanity as of insanity.

[8] Annales Medico Psychologiques, 1844. [9] Jour. of Ment. Sci., 1896. [10] Jour. of Ment. Sci., April, 1864. [11] Psychological Medicine. [12] Natural Inheritance.

